# Effects of *n*-3 Polyunsaturated Fatty Acids (ω-3) Supplementation on Some Cardiovascular Risk Factors with a Ketogenic Mediterranean Diet

**DOI:** 10.3390/md13020996

**Published:** 2015-02-13

**Authors:** Antonio Paoli, Tatiana Moro, Gerardo Bosco, Antonino Bianco, Keith A. Grimaldi, Enrico Camporesi, Devanand Mangar

**Affiliations:** 1 Department of Biomedical Sciences, University of Padova, 35131 Padova, Italy; E-Mails: tatiana.moro.phd@gmail.com (T.M.); gerardo.bosco@unipd.it (G.B.); 2 Sport and Exercise Sciences Research Unit, University of Palermo, 90144 Palermo, Italy; E-Mail: antonino.bianco@unipa.it; 3 Biomedical Engineering Laboratory, Institute of Communication and Computer Systems, National Technical University of Athens, Athens 15773, Greece; E-Mail: keith.grimaldi@gmail.com; 4 Department of Surgery, University of South Florida, Tampa, FL 33620, USA; E-Mail: ecampore@health.usf.edu; 5 TEAMHealth, Tampa, FL 33606, USA; E-Mail: dmangar1@gmail.com; 6 Tampa General Hospital, Tampa, FL 33606, USA

**Keywords:** inflammatory cytokines, ketogenic diet, weight loss, cardiovascular risk factors, *n*-3 polyunsaturated fatty acids, omega-3

## Abstract

Background: the ketogenic diet (KD) has become a widely used nutritional approach for weight loss. Some of the KD’s positive effects on metabolism and cardiovascular risk factors are similar to those seen after *n*-3 polyunsaturated fatty acids (ω-3) supplementation. We hypothesized that a ketogenic Mediterranean diet with phytoextracts combined with ω-3 supplementation may have increased positive effects on cardiovascular risk factors and inflammation. Methods: We analyzed 34 male overweight subjects; aged between 25 and 65 years who were overall healthy apart from overweight. The subjects followed a ketogenic diet protocol for four weeks; with (KDO3) or without (KD) ω-3 supplementation. Results: All subjects experienced a significant loss of body weight and body fat and there was no significant differences between treatment (body weight: KD—4.7 kg, KDO3—4.03 kg, body fat KD—5.41 kg, KDO3—5.86 kg). There were also significant decreases in total cholesterol, LDL-c, and glucose levels. Triglycerides and insulin levels decreased more in KDO3 *vs.* KD subjects, with a significant difference. All the investigated inflammatory cytokines (IL-1β, IL-6, TNF-α) decreased significantly in KDO3 subjects whilst only TNF-α showed a significant decrease in KD subjects over the 12 month study period. No significant changes were observed in anti-inflammatory cytokines (IL-10 and IL-1Ra), creatinine, urea and uric acid. Adiponectin increased significantly only in the KDO3 group. Conclusions: ω-3 supplementation improved the positive effects of a ketogenic Mediterranean diet with phytoextracts on some cardiovascular/metabolic risk factors and inflammatory state.

## 1. Introduction

In recent years there has been an increasing amount of evidence suggesting that a ketogenic diet, apart from its benefits for weight loss [1,2], also has a beneficial effect on the symptoms of neurological diseases [3], diabetes [4] and other diseases [5]. The use of ketogenic diets is widely accepted by physicians in some well-defined fields such as pediatric pharmacoresistant epilepsy [6] or Glut1 deficiency syndrome [7]. However there are still some concerns about its effects on health outcomes on blood lipid profiles and cardiovascular risk factors [8], even though the majority of published studies do not support such concerns [1]. The majority of recent studies clearly demonstrate that the reduction of carbohydrate intake leads to a *physiological* ketosis (that must be distinguished from the pathological ketosis arising from diabetes, as stated by Hans Krebs in 1966 [9]) that improves blood lipid profiles [10,11,12,13,14,15]. The effects of KDs seem to be more evident on levels of blood triglycerides [16] but they also have positive effects on the reduction of total cholesterol and an increase in HDL [17,18]. Moreover KDs have been reported to increase the size and volume of LDL-C particles [17], which leads to a reduced cardiovascular disease risk (small LDL particles have a higher atherogenicity).

The marine *n*-3 polyunsaturated fatty acids (ω-3) eicosapentaenoic acid (EPA) and docosahexaenoic acid (DHA) are present in oily fish and, following the publication, almost 30 years ago, of the cornerstone study on cardiovascular mortality rate in Greenland Eskimos [19], they have been available as commercial supplements. Whilst many health benefits have been attributed to the supplements, mainly on the cardiovascular system, no conclusive data has yet been published, e.g., a recent meta-analysis by Rizos and co-workers [20] reported no significant effects ofω-3 (EPA–DHA) supplementation on the incidence of cardiovascular events such as stroke or myocardial infarction. On the other hand a large number of studies suggest beneficial effects of *n*-3 polyunsaturated fatty acids (*n*-3 PUFA) supplementation on cardiovascular risk factors. Based on the latter evidence current nutritional guidelines recommend the use of *n*-3 PUFA in the general population and in subjects with cardiac disorders [21].

There are several possible mechanisms that may mediate the positive effects of ω-3 on the cardiovascular system, these include mitochondrial biogenesis [22], changes in tissue levels of endocannabinoids that influence blood pressure [23], improvements in vascular endothelial cell function [24], decreased inflammation [25], and inhibition of phosphatidic acid phosphodyrolase and diacylglycerol acyltransferase, leading to decreased TG synthesis [26].

Thus, considering the overlapping effects, we investigated the possible associations between a relatively high fat/very low carbohydrate ketogenic diet and ω-3 supplementation and how this association may affect some cardiovascular risk factors. This is, to our best knowledge, the first study aimed at evaluating the effects of ω-3 supplementation during a ketogenic diet in overweight subjects.

## 2. Results

Of the 38 subjects (See Table 1 for subjects’ anthropometric characteristics), 35 finished the study (18 in KD and 17 in KDO3), two subjects withdrew due to personal reasons (KDO3), one withdrew because he did not like the diet (KD), and one further subject failed to return for the follow-up visits and was dropped from the study (KDO3 group). Thus 34 subjects were included in the final analysis (18 KD, 16 KDO3). Both groups showed a significant decrease in body mass index (BMI), fat mass (FM) with similar results in both KD and KDO3 whilst lean body mass (LBM) showed no significant changes. Both groups showed a decrease in total cholesterol without any significant difference according to treatment, low-density lipoprotein cholesterol (LDL-c) decreased in both groups with a greater decrease, although not significant, in the KD group. High-density lipoprotein cholesterol (HDL-c) showed no significant change. Triacyglycerol (TG) decreased significantly in both groups with a greater, significant decrease in KDO3. Insulin decreased significantly in both groups, but to a greater, significant extent in KDO3. Adiponectin increased significantly only in the KDO3 group. Interleukin 1 beta (IL-1β) and interleukin 6 (IL-6) levels decreased significantly only in the KDO3 group whilst tumor necrosis factor alpha (TNF-α) decreased in both groups but was significantly greater in the omega 3 supplemented group. Anti-inflammatory cytokine interleukin-1 receptor antagonist (IL-1Ra) and interleukin 10 (IL-10) showed no significant changes. Results are summarized in Table 2.

**Table 1:** Subjects’ basal characteristics (data are expressed as mean and SD).

	KD Group	KDO3 Group
Age (years)	56.3 ± 5.1	58.1 ± 6.0
Height (cm)	177.2 ± 4.7	180 ± 7.9
Weight (kg)	92.25 ± 12.53	94.5 ± 15.20
BMI	29.34 ± 2.40	29.17 ± 2.37
LBM (kg)	58.06 ± 14.68	58.87 ± 18.19
FM (kg)	34.19 ± 3.93	35.93 ± 6.76

**Table 2:** Changes in anthropometric and blood parameters at baseline (pre) and after ketogenic diet treatment (post) without (KD) or with (KDO3) omega 3 supplementation. Values are expressed as mean and standard deviation.

	KD Pre	KD Post	Δ If Pre-Post Tukey’s t	KDO3 Pre	KDO3 Post	Δ CTR Pre-Post Tukey’s t	Between Treatment ANOVA t
Weight (kg)	92.25 ± 12.53	87.55 ± 10.03	*p* < 0.05	94.5 ± 15.2	90.48 ± 12.71	*p* < 0.05	n.s.
BMI	29.34 ± 2.41	27.87±1.70	*p* < 0.05	29.17 ± 2.38	27.96 ± 1.80	*p* < 0.05	n.s.
LBM (kg)	58.06 ± 14.68	58.77±12.02	n.s.	58.87 ± 18.19	60.41 ± 15.09	n.s.	n.s.
FM (kg)	34.19 ± 3.93	28.78 ± 4.37	*p* < 0.005	35.93 ± 6.76	30.06 ± 5.09	*p* < 0.005	n.s.
IL-6 (pg/mL)	6.13 ± 0.81	4.38 ± 1.05	n.s.	6.55 ± 1.41	3.65 ± 1.27	*p* < 0.05	*p* < 0.05
IL-1β (pg/mL)	2.77 ± 0.85	1.88 ± 0.5	n.s.	3.05 ± 0.51	1.2 ± 0.73	*p* < 0.05	*p* < 0.05
TNF-α (pg/mL)	5.63 ± 0.82	4.1 ± 0.73	*p* < 0.05	5.23 ± 0.67	2.8 ± 0.34	*p* < 0.001	*p* < 0.05
IL-1Ra	287 ± 101.54	307 ± 89.57	n.s.	299 ± 72.36	301 ± 88.95	n.s.	n.s.
IL-10	5.10 ± 3.8	6.25 ± 2.25	n.s.	4.7 ± 4.3	5.15 ± 3.37	n.s.	n.s.
Insulin (μIU/mL)	9.3 ± 2.00	7.4 ± 0.90	*p* < 0.05	11.1 ± 1.90	6.3 ± 1.10	*p* < 0.05	*p* < 0.05
Glucose (mg/dL)	103.5 ± 5.30	85.3 ± 4.70	*p* < 0.05	107.8 ± 6.30	82.1 ± 7.20	*p* < 0.05	n.s.
Adiponectin (μg/mL)	7.18 ± 0.67	7.83 ± 0.39	n.s.	6.55 ± 1.02	7.85 ± 0.37	*p* < 0.05	*p* < 0.05
Tot Chol (mg/dL)	217.25 ± 15.84	201.28 ± 6.79	*p* < 0.05	222.39 ± 6.10	204.52 ± 9.78	*p* < 0.05	n.s.
HDL-c (mg/dL)	36.28 ± 2.23	39.25 ± 1.37	n.s.	39.55 ± 2.99	40.25 ± 2.63	n.s.	n.s.
LDL-c (mg/dL)	133.41 ± 15.86	123.60 ± 7.99	*p* < 0.05	136.98 ± 7.06	127.56 ± 7.19	P < 0.05	n.s.
TG (mg/dL)	237.81 ± 20.26	197.27 ± 6.1	*p* < 0.05	230.79 ± 25.66	185.54 ± 9.64	P < 0.05	*p* < 0.05
Urea (mg/dL)	33.91 ± 7.18	35.22 ± 6.35	n.s.	35.18 ± 6.58	36.33 ± 7.35	n.s.	n.s.
Uric acid (mg/dL)	4.78 ± 1.03	4.89 ± 0.90	n.s.	5.05 ± 1.22	4.93 ± 0.78	n.s.	n.s.
Creatinine (mg/dL)	0.78 ± 0.15	0.76 ± 0.13	n.s.	0.75 ± 0.17	0.73 ± 0.15	n.s.	n.s.

## 3. Discussion

Our results confirm the positive effects of a Mediterranean ketogenic diet with phytoextracts on body composition and some cardiovascular risk factors. Supplementation with ω-3 fatty acids further improved the effects of KD on TG, insulin and adiponectin and in addition decreased inflammatory mediators such as IL-1β, IL-6, TNF-α. To the best of our knowledge there has only been one study published so far which investigated the synergy between a ketogenic diet and ω-3 supplementation, however this was performed in children with drug-resistant epilepsy [27].

Following the discovery of the low cardiovascular mortality rate in Greenland Eskimos [19], many studies have been published on the possible role of *n*-3 PUFAs in the prevention of CVD. The results have been inconsistent, several studies show promising evidence regarding the effects on primary and secondary prevention of CVD [28,29,30] while others found no effects [20]. Notwithstanding these conflicting results [30] and the further research required, the current guidelines, based on the risk/benefit findings, recommend two servings of fatty fish per week [21]. This is consistent with the difficulty of conducted studies on nutritional interventions with CVD incidence or mortality as the end point. The recommendations are instead influenced by studies where omega 3 has been shown, consistently, to improve biomarker risk factors such as TG, LDL, and HDL [31]. A reduction of total cholesterol is also a consistent finding of research on the effect of KDs [1,10,15,17,32]. The evidence is that these effects are mediated by the facilitatory action of insulin on HMGCoA reductase (which is the target for statins) and the opposite inhibitory effects of cholesterol and fats; *i.e.*, insulin increases the endogenous production of cholesterol which, via feedback inhibition of the enzyme HMGCoA reductase, cholesterol, has an opposite effect on endogenous cholesterol production [33]. The effect of KD on insulin is likely to be a factor for the reported efficient decrease of LDL cholesterol and TG, and the increase in HDL cholesterol. In the current study the data show that the addition of ω-3 supplementation to the KD did not further improve cholesterol values but did demonstrate a greater decrease of TG. While the mechanisms of KD, via insulin lowering, on cholesterol are well documented, the TG and cholesterol lowering mechanisms of ω-3, on the contrary, are not completely understood. A possible hypothesis is that ω-3 are able to reduce FA availability (which are substrates for the TG) or decrease the activity of diacylglycerol acyltransferase or phosphatidic acid phosphodyrolase, the key enzymes that synthesize TGs. Moreover, EPA increases LPL-mediated extracellular lipolysis that leads to an increased VLDL clearance from the circulation and EPA/DHA increases the blood clearance of TG-rich particles [34].

It has been suggested that ω-3 could act through some surface or intracellular receptors involved in transcriptional events. These receptors are peroxisome proliferator-activated receptors (PPARs), hepatocyte nuclear factor alpha 4 and liver X receptors all of which are under the control of sterol receptor element binding protein-1c (SREBP-1), a regulator of lipogenesis. A key element in this complex picture is PPAR-α, a component of the transcription factor (PPARS) family involved in gene expression regulation [35]. In particularly PPAR-α is the isoform preferentially expressed in the liver that enhances beta oxidation in both liver and skeletal muscles. Another isoform, the PPAR-γ, is preferentially expressed in adipocytes and inflammatory cells, and is involved in inflammatory pathways and insulin sensitivity. Thus PPAR activation might be a candidate for the mechanisms involved in the decrease of TGs and inflammation markers and in the increase in insulin sensitivity effects of ω-3. The activation of PPARs by omega 3 results in an increased FA oxidation in the liver and skeletal muscle that leads to a reduced TG availability for the VLDL synthetic pathway [36]. The small increase of LDL after ω-3 supplementation observed in some studies is compensated by the increase in the diameter of LDL particles (that reduces their atherogenic action) [17,37].

Recently, Rossmeisl *et al.* demonstrated that ω-3 supplementation in mice decreased hepatic and plasma cholesterol levels through an inhibition of genes involved in hepatic cholesterol synthesis and an increased gene expression of sterol transporters involved in the efflux of cholesterol into the intestinal and biliary lumen for faecal excretion [38]. The authors suggest that the inhibition of lipogenic and cholesterol biosynthesis pathways could be due to the inhibition of the activity of mitochondrial citrate carrier.

Other noteworthy observations should be made regarding the treatment which affected variation in the cytokines. Some research studies have shown an anti-inflammatory effect of KDs at least at the CNS level [39] but also at a more general level [40]. A study by Forsythe and colleagues demonstrated a higher anti-inflammatory effect of a KD compared to a low fat diet [41]. These findings are not surprising considering that the metabolic state imposed by KD can be compared to prolonged fasting which is reportedly able to reduce systemic inflammation [42]. It has been suggested that the anti-inflammatory effects of carbohydrate restriction could be mediated via down-regulation of NF-κB expression [41].

On the other hand the relationship between *n*-3 PUFA and inflammation is well documented [43] and low levels of blood ω-3 are related to inflammation biomarkers [44]. Even though some researches have shown a weak association between ω-3 supplementation and inflammatory cytokines in healthy men [45] and others have provided some conflicting information [46], the majority of available evidence suggests a possible omega-3 PUFA anti-inflammatory action [47,48].

Our data suggests that a ketogenic Mediterranean diet with phytoextracts is able to reduce low-grade systemic inflammation and that its effect is enhanced by ω-3 supplementation. The exact mechanism by which ω-3 fatty acids exert their anti-inflammatory effect is not clearly understood and many mechanisms have been proposed. One candidate is EPA and DHA binding with G protein-coupled receptor 120 (GPR120): Once activated the GPR120 form co-localizes with β-arrestin2 in the plasma membrane which is then internalized into the cytoplasm, as a complex. The reaction between this complex and TAB1 leads to the inhibition of TAB1 association with TAK1, blocking the activation of inflammatory mediators pathways [49]. Furthermore, it has also been demonstrated that EPA and DHA can inhibit the TLR-4 signaling pathway [50]. Two mechanisms seem to be involved in TLR-4 inhibition: The first, the reduction of ROS production (necessary for TLR-4 signaling) through the down-regulation of nicotinamide adenine dinucleotide phosphase-oxidase (NADPH oxidase) production [51], an important ROS source, and secondly, the incorporation of DHA into lipid membranes that disturbs the translocation of TLR-4 into lipid rafts [52]. The altered TLR-4 signaling pathways lead to the inhibition of NF-κB (as do ketogenic diets) and consequently an inflammatory down-regulation response. The reduction of fasting glucose and insulin levels is an expected and logical outcome of very low carbohydrate ketogenic diets. After a few days of carbohydrate restriction, blood insulin level is reduced whilst blood glucose stabilizes at low, but physiological levels [32]. Our results showed that ω-3 supplementation had a greater lowering effect on insulin compared to the non-intervention group. Several studies have confirmed that omega-3 might interfere with insulin secretion, leading to a decrease in circulating insulin levels and a concomitant rise in blood glucose [53]. This effect on insulin sensitivity may be mediated by increased adiponectin production induced by EPA and DHA [54]—Adiponectin stimulates AMPK in the muscle and then the downstream oxidative pathways [55]. Also fat metabolism is positively affected by omega-3 through the AMPK pathway, and moreover the increased lipids profile could contribute to an improved insulin control. The significant increase of adiponectin in KDO3 could partially explain the differences in insulin levels between the two groups.

## 4. Materials and Methods

### 4.1. Subjects

Forty three healthy male subjects were recruited by means of flyers. Of the 43 interested individuals, 38 were deemed eligible to participate to the study (see Table 1) according to a preliminary questionnaire and to a subsequent body mass index (BMI) assessment. Key inclusion criteria were as follows: Age 30–65 years; BMI over 25 kg/m^2^; stable weight for 3 months prior to the beginning of the study (*i.e.*, less than 5 kg loss or weight gain); non-diabetic; no history of cardiovascular disease; no previous ω-3 assumption; sedentary; no history of bariatric surgery; and not taking weight loss, lipid or glucose lowering medications. Six subjects of the KD group and four of the KDO3 were taking antihypertensive drugs. The experimental protocol was approved by the local Departmental Ethical Committee. All volunteers gave informed consent to participate in the trial.

### 4.2. Experimental Design and Randomization

A 4-week, randomized, controlled, parallel-arm feeding trial was implemented to test the effects of a KD with or without ω-3 supplementation on blood parameters and weight loss. Subjects were divided into ketogenic diet without supplementation (KDA, 19 subjects) and ketogenic diet plus ω-3 (KDO3, 19 subjects).

### 4.3. Diet Protocol

The KD consisted of a commercial protocol (KEMEPHY: Ketogenic Mediterranean diet with Phytoextracts, Tisanoreica^®^, Gianluca Mech SpA, Asigliano Veneto, VI, Italy) that had been previously described [13,15]. The diet consisted of a very low carbohydrate ketogenic diet with the use of some phytoextracts. The diet was explained to all subjects by a qualified dietician during an individual visit. During KEMEPHY, subjects almost totally excluded carbohydrates. A detailed menu containing permitted and non-permitted foods was provided to each participant, along with the components of the ketogenic Mediterranean diet with phytoextracts (see Table 3 for details). The consumed diet was primarily made of beef and veal, poultry, fish, raw and cooked green vegetables without restrictions, cold cuts (dried beef, *carpaccio* and cured ham), eggs and seasoned cheese (e.g., parmesan). The allowed drinks were infused tea, *moka* coffee and herbal extracts. The foods and drinks that subjects avoided included alcohol, bread, pasta, rice, milk, yogurt, soluble tea, and barley coffee. In addition, to facilitate the adherence to the nutritional regime, a variety of special meals based on protein and fibers was given to each subject. These meals (Tisanoreica^®^, Gianluca Mech SpA, Asigliano Veneto, VI, Italy) which were composed of high quality proteins (equivalent to 18 g/portion) and virtually zero carbohydrates (excepting mimics of their taste) were included in the standard ration [15,16,32]. Subjects also consumed some specific herbal extracts described in Table 4 [15]. In addition, during the ketogenic diet periods, subjects assumed each morning one caplet of a multivitamin-mineral supplement—Containing magnesium19 mg, calcium 16 mg, phosphorus 8 mg, zinc 4.5 mg, iron 4.62 mg, manganese 1 mg, potassium 0.5 mg, copper 0.4 mg, chromium 28.55 μg, selenium 4 μg, niacin 10 mg, beta carotene 1.8 mg, folic Acid 66 μg, biotin 30 μg, vitamin C 19.8 mg, vitamin E3.3 mg, pantothenic acid 1.98 mg, vitamin B6 0.66 mg, vitamin B2 0.53 mg, vitamin B1 0.426 mg, vitamin D3 1.65 μg, vitamin B12 0.33 μg (Multivitaminico Balestra e Mech, Gianluca Mech SpA, Asigliano Veneto, VI, Italy).

**Table 3:** Characteristics of KEMEPHY diet (data are expressed as mean and SD).

	KD Group	KDO3 Group
Kcal/day	1187 ± 89	1218 ± 105
Protein (% total daily Kcal)	43.4 ± 3.2	43.2 ± 4
Fat (% total daily Kcal)	45.8 ± 4	53.7 ± 5.2
Carbohydrate (% total daily Kcal)	10.8 ± 2.1	10.5 ± 1.8
Protein(g/day)	128.8 ± 10	131.6 ± 8.2
Fat(g/day)	60.5 ± 8.9	62.6 ± 7.6
Carbohydrates(g/day)	32 ± 1.8	32 ± 2.1

**Table 4:** Plant extracts used during KEMEPHY diet [15].

Plant Extracts	mL/day	*Composition*
Extracts A, mL/day	20	Durvillea antarctica, black radish, mint, liquorice, artichoke, horsetail, burdock, dandelion, rhubarb, gentian, lemon balm, chinaroot, juniper, spear grass, elder, fucus, anise, parsley, bearberry, horehound
Extracts B, mL/day	20	Serenoa, red clover, chervil, bean, elder, dandelion, uncaria, equisetum, horehound, rosemary
Extracts C, mL/day	50	Horsetail, asparagus, birch, cypress, couch grass, corn, dandelion, grape, fennel, elder, rosehip, anise
Extracts D, mL/day (only weeks 1 and 2)	40	Eleuthero, eurycoma longifolia, ginseng, corn, miura puama, grape, guaranà, arabic coffee, ginger

During the experimental protocol, the subjects enrolled in the KDO3 group also ingested two caplets of omega 3 from krill sources [56] (Euphausia superba): 57.5 mg EPA, 32.5 DHA, astaxantine 25 microg, total ω-3 115 mg, sea phospholipids 200 mg (Omega-T, Gianluca Mech SpA, Asigliano Veneto, VI, Italy). A telephone interview was performed by one of the researchers in order to verify adhesion to the assigned diet.

### 4.4. Measurements

Dietary adherence was measured by a validated 7-day food diary [57] and analyzed by Dietnext^®^ (Caldogno, Vicenza, Italy) software. Diet composition is shown in Table 3. Subjects underwent blood analysis, anthropometric measurements, and body composition analysis at the start and the end of the protocol. Weight was measured to the nearest 0.01 kg using an electronic scale, and height to the nearest 0.01 m using a Harpenden portable stadiometer. Body composition was assessed using bioelectrical impedance analysis (BIA Akern Bioresearch, Pontassieve, FI, Italy) which is a non-invasive and a portable method for the estimation of fluid compartments, fat and fat-free mass in healthy subjects. Bioelectrical impedance analysis was chosen because it is a reliable, safe, convenient, and non-invasive method that makes it a useful procedure to be deployed in the monitoring routine of body composition during the ketogenic diet [58]. Fasting venous blood samples were collected pre- and post-intervention for the total cholesterol (CHOL-t), triacyglycerol (TG), high-density lipoprotein cholesterol (HDL-c), low-density lipoprotein cholesterol (LDL-c), glucose, blood urea, uric acid, creatinine, insulin and adiponectin, interleukin 1 beta (IL-1β), interleukin 6 (IL-6), tumor necrosis factor alpha (TNF-α), interleukin-1 receptor antagonist (IL-1Ra) and interleukin 10 (IL-10). Blood was collected in EDTA treated in vacutainer tubes. A separate blood sample was clotted and serum analyzed for total cholesterol and triacylglycerols by photometric assay. HDL cholesterol was determined using a homogenous enzyme immunoassay. Plasma glucose was determined by GLUCO-QUANT glucose/hexokinase test (Roche Diagnostics, Birch Run, MI, USA) using Modular Analyzer (Roche Diagnostics). Insulin was measured by the Enzyme ImmunoAssay test (DPC, Los Angeles, CA, USA) using Immulite 2000 (Siemens Medical Solutions, Malvern, PA, USA). Plasma adiponectin were measured using an ELISA kit (Immunodiagnostik, AG, Stubenwald-Allee 8a, D 64625 Bensheim, Germany). Plasma urea nitrogen was measured using an enzymatic (urease), colorimetric method. Creatinine was measured calorimetrically using the picric acid assay, and uric acid was determined using a modified Trinder peroxide assay. LDL-c fraction was calculated from Friedewald’s formula: LDL-c = TC–HDL-c–(TG/5). IL-6 and TNF-α, IL-1β, IL-1Ra and IL-10 were also measured with Quantikine HS Immunoassay Kit (R & D Systems, Minneapolis, MN, USA).

### 4.5. Statistical Analysis

The effect of the diet intervention and supplementation was assessed using a two-way repeated measure ANOVA (time *vs.* nominal variables KD and KDO3 *vs.* measures). When significant effects were found between and/or within, the *post hoc* analysis was performed using Tukey’s test. An alpha level of *p* < 0.05 was used to denote a significant effect. Kolmogorov-Smirnov tests were used to assess the normality of the data. Mauchley’s test of sphericity assessed the homogeneity of variance for the data. Subjects were randomly assigned to the two experimental groups. All statistical analyses were performed using the software package GraphPad Prism version 6.00 for Mac, GraphPad Software, San Diego, CA, USA. Values are represented as means and standard deviations (SD).

## 5. Conclusions

Our data confirms the positive effects of a Mediterranean ketogenic diet with phytoextracts on weight loss, body composition, blood lipid profiles, and inflammatory markers. Moreover, our results suggest that omega 3 supplementation does not alter the effects of a KD on body weight reduction, fat loss, or blood cholesterol profile improvement, but rather it increases further the KD’s positive effects on inflammatory markers, adiponectin, insulin, and blood triglycerides. These conclusions are consistent with previous observations on the anti-inflammatory effects of carbohydrate reduction but for the first time we have demonstrated the usefulness of Ω-3 supplementation during a ketogenic diet. This kind of supplementation may optimize the positive effects of KDs on some cardiovascular risk markers and on obesity-related chronic low grade inflammation.

## Abbreviations

IL-1β: interleukin 1 beta
IL-1Ra: interleukin-1 receptor antagonist
IL-10: interleukin 10
IL-6: interleukin 6
TNF-α: tumor necrosis factor alpha
